# Private sector opportunities and threats to achieving malaria elimination in the Greater Mekong Subregion: results from malaria outlet surveys in Cambodia, the Lao PDR, Myanmar, and Thailand

**DOI:** 10.1186/s12936-017-1800-5

**Published:** 2017-05-02

**Authors:** ACTwatch Group, Louis Akulayi, Angela Alum, Andrew Andrada, Julie Archer, Ekundayo D. Arogundade, Erick Auko, Abdul R. Badru, Katie Bates, Paul Bouanchaud, Meghan Bruce, Katia Bruxvoort, Peter Buyungo, Angela Camilleri, Emily D. Carter, Steven Chapman, Nikki Charman, Desmond Chavasse, Robyn Cyr, Kevin Duff, Gylsain Guedegbe, Keith Esch, Illah Evance, Anna Fulton, Hellen Gataaka, Tarryn Haslam, Emily Harris, Christine Hong, Catharine Hurley, Whitney Isenhower, Enid Kaabunga, Baraka D Kaaya, Esther Kabui, Beth Kangwana, Lason Kapata, Henry Kaula, Gloria Kigo, Irene Kyomuhangi, Aliza Lailari, Sandra LeFevre, Megan Littrell, Greta Martin, Daniel Michael, Erik Monroe, Godefroid Mpanya, Felton Mpasela, Felix Mulama, Anne Musuva, Julius Ngigi, Edward Ngoma, Marjorie Norman, Bernard Nyauchi, Kathryn A. O’Connell, Carolyne Ochieng, Edna Ogada, Linda Ongwenyi, Ricki Orford, Saysana Phanalasy, Stephen Poyer, Justin Rahariniaina, Jacky Raharinjatovo, Lanto Razafindralambo, Solofo Razakamiadana, Christina Riley, John Rodgers, Andria Rusk, Tanya Shewchuk, Simon Sensalire, Julianna Smith, Phok Sochea, Tsione Solomon, Raymond Sudoi, Martine Esther Tassiba, Katherine Thanel, Rachel Thompson, Mitsuru Toda, Chinazo Ujuju, Marie-Alix Valensi, Vamsi Vasireddy, Cynthia B. Whitman, Cyprien Zinsou, Sochea Phok, Saysana Phanalasy, Si Thu Thein, Asawin Likhitsup

**Affiliations:** 1 Population Services International, 1120 19th St NW, Suite 600, Washington, DC 20036 USA; 2 Population Services Khmer, 29 334 St, Boeung Keng Kang, P. O. Box 258, Phnom Penh, Cambodia; 3 Population Services International Lao PDR, T4 Road Unit 16, Donkai Village, P. O. Box 8723, Vientiane, Lao People’s Democratic Republic; 4 Population Services International Myanmar, 16 West Shwe Gone Dine 4th St, Bahan Township, Yangon, Myanmar; 5 108/210 Siphraya River View Condo, Yotha Rd, Sampanthawong, Bangkok, 10100 Thailand

**Keywords:** Anti-malarial, Private sector, Greater Mekong Subregion (GMS), First-line, Second-line, Market share, Availability, RDT

## Abstract

**Background:**

The aim of this paper is to review multi-country evidence of private sector adherence to national regulations, guidelines, and quality-assurance standards for malaria case management and to document current coverage of private sector engagement and support through ACTwatch outlet surveys implemented in 2015 and 2016.

**Results:**

Over 76,168 outlets were screened, and approximately 6500 interviews were conducted (Cambodia, N = 1303; the Lao People’s Democratic Republic (PDR), N = 724; Myanmar, N = 4395; and Thailand, N = 74). There was diversity in the types of private sector outlets providing malaria treatment across countries, and the extent to which they were authorized to test and treat for malaria differed. Among outlets stocking at least one anti-malarial, public sector availability of the first-line treatment for uncomplicated *Plasmodium falciparum* or *Plasmodium vivax* malaria was >75%. In the anti-malarial stocking private sector, first-line treatment availability was variable (Cambodia, 70.9%; the Lao PDR, 40.8%; Myanmar *P. falciparum* = 42.7%, *P. vivax* = 19.6%; Thailand *P. falciparum* = 19.6%, *P. vivax* = 73.3%), as was availability of second-line treatment (the Lao PDR, 74.9%; Thailand, 39.1%; Myanmar, 19.8%; and Cambodia, 0.7%). Treatment not in the National Treatment Guidelines (NTGs) was most common in Myanmar (35.8%) and Cambodia (34.0%), and was typically stocked by the informal sector. The majority of anti-malarials distributed in Cambodia and Myanmar were first-line *P. falciparum* or *P. vivax* treatments (90.3% and 77.1%, respectively), however, 8.8% of the market share in Cambodia was treatment not in the NTGs (namely chloroquine) and 17.6% in Myanmar (namely oral artemisinin monotherapy). In the Lao PDR, approximately 9 in 10 anti-malarials distributed in the private sector were second-line treatments—typically locally manufactured chloroquine. In Cambodia, 90% of anti-malarials were distributed through outlets that had confirmatory testing available. Over half of all anti-malarial distribution was by outlets that did not have confirmatory testing available in the Lao PDR (54%) and Myanmar (59%). Availability of quality-assured rapid diagnostic tests (RDT) amongst the RDT-stocking public sector ranged from 99.3% in the Lao PDR to 80.1% in Cambodia. In Cambodia, the Lao PDR, and Myanmar, less than 50% of the private sector reportedly received engagement (access to subsidized commodities, supervision, training or caseload reporting), which was most common among private health facilities and pharmacies.

**Conclusions:**

Findings from this multi-country study suggest that Cambodia, the Lao PDR, Myanmar, and Thailand are generally in alignment with national regulations, treatment guidelines, and quality-assurance standards. However, important gaps persist in the private sector which pose a threat to national malaria control and elimination goals. Several options are discussed to help align the private sector anti-malarial market with national elimination strategies.

**Electronic supplementary material:**

The online version of this article (doi:10.1186/s12936-017-1800-5) contains supplementary material, which is available to authorized users.

## Background

Malaria elimination is the goal of all countries in the Greater Mekong Subregion (GMS), with accelerated achievement a priority due to the emergence and spread of artemisinin drug resistant parasites. The World Health Organization (WHO) Strategy for Malaria Elimination in the GMS (2015–2030) sets a target of malaria elimination in all GMS countries by 2030 and *Plasmodium falciparum* malaria by 2025 [1]. Appropriate case management of all suspected malaria cases, including early confirmatory diagnosis and prompt treatment with effective, first-line anti-malarial medicines, is essential to achieving the WHO elimination goals.

National programmes across the region have defined National Treatment Guidelines (NTG) stipulating the use of different first- and second-line treatments for uncomplicated and severe malaria (Table 1) for any *Plasmodium* species infection. These guidelines vary by country in part due to the need to continually update guidelines based on the latest evidence regarding anti-malarial drug tolerance, therapeutic efficacy, and resistance [2].

**Table 1:** National Treatment Guidelines Cambodia, Lao PDR, Myanmar, and Thailand

Country	Year	First-line *Pf*	First-line *Pv*	First-line severe malaria	Second-line
Cambodia	2014	Fixed-dose combination (FDC) artesunate-mefloquine (ASMQ) + primaquine or dihydroartemisinin-piperaquine + primaquine (DHA-PPQ)		Artesunate Intravenous (IV)/Intramuscular (IM) or Artemether IV/IM	*Pf*/*Pv*: Quinine + doxycycline or tetracycline
Lao PDR	2013	Artemether–Lumefantrine (AL) + primaquine		Artesunate IV/IM	*Pf*: Quinine + doxycycline *Pv*: Chloroquine Severe: Quinine IV/IM
Myanmar	2012	AL or ASMQ or DHA PPQ + primaquine^a^	Chloroquine + primaquine	Artesunate IV/IM	*Pf*: Alternative first-line artemisinin-based combination medicines or artesunate + either doxycycline, tetracycline, or clindamycin Severe: Artemether IV/IM or Quinine IV/IM
Thailand	2014	ASMQ or DHA PPQ^b^ + primaquine	Chloroquine + primaquine	Artesunate IV/IM	*Pf*: Quinine + doxycycline, or artesunate + doxycycline or clindamycin *Pv*: Chloroquine Severe: Quinine IV/IM

^a^In late 2015, the Myanmar NTGs changed to AL + primaquine

^b^In late 2015, the Thailand malaria treatment guidelines changed in eight provinces to stipulate use of DHA PPQ, with a single dose of primaquine (30 mg) on day 1

Achieving universal coverage with quality-assured diagnostics and anti-malarials requires three channels of service delivery to be considered: public, private, and community-based [1]. It is acknowledged by the WHO that the optimal mix of these channels will vary between and within countries and in elimination settings, and that the roles for each channel should be reviewed and defined, depending on the country situation and local conditions, to ensure optimal case management, surveillance, and reporting in all areas.

In the GMS, the role of the private sector has been recognized as an important source of anti-malarial treatment in many countries, including Cambodia, Myanmar, and the Lao People’s Democratic Republic (PDR). Supply-side surveys have illustrated how most anti-malarial medicines are distributed through the private sector [3, 4], and these findings are complemented by population-based surveys from these countries which illustrate that febrile patients commonly seek treatment in the private sector [5–7]. While the private sector is relevant across the region, the specific types of outlets that provide malaria testing and treatment differs by country. In addition, national policies vary with respect to the specific providers and outlet types that are authorized to test for and treat malaria (Table 2). In the Lao PDR, all private for-profit health facilities and pharmacies are permitted to provide malaria testing and treatment, whereas in Cambodia only private health facilities and registered pharmacies in the Public–Private Mix (PPM) programme are authorized to test and treat. In grocery stores and general retailers, and among itinerant drug vendors, the sale of anti-malarials is prohibited by national authorities in Cambodia, the Lao PDR, and Thailand but not in Myanmar. In Thailand, the private sector is almost completely prohibited to provide anti-malarials or confirmatory testing, and only certain private hospitals are permitted to provide testing and treatment on a case-by-case basis.

**Table 2:** Outlet type definitions

Outlet type	Description	Authorized to test and treat			
		Cambodia	Lao PDR	Myanmar	Thailand
Public sector					
Public health facility	Public health facilities include government health facilities as well as private not-for-profit facilities	×	×	×	×
Community health worker	Community health workers (CHW) are community-based volunteers typically linked with government or non-government not-for-profit organizations and are equipped with anti-malarial treatment and malaria RDT. CHW receive formal training on malaria case management	×	×	×	×
Private sector					
Private health facility	Private for-profit health laboratories, clinics, and hospitals that provide healthcare to the general public	×	×	×	
Pharmacy	Pharmacies are typically licensed and regulated by a national regulatory authority and are staffed by pharmacists and qualified health practitioners	×	×	×	
Drug store	Drug stalls in rural markets and shops that primarily sell medicines. These outlets are not guaranteed to be staffed by qualified health dispensers/practitioners and are not licensed by a national regulatory authority. Drug stores are not found in Myanmar				
General retailer	General retailers are grocery stores and village shops that sell fast-moving consumer goods, food, and provisions			×	
Itinerant drug vendor	Mobile drug vendors found primarily in rural areas, typically working within the radius of their home or travelling to nearby villages to deliver medicines. They are not registered with any national regulatory authority			×	

Although the private sector plays a significant role in malaria case management throughout these countries, several challenges have been noted with the performance of this sector. There may be a lack of knowledge among providers on where to refer patients with more severe conditions and limited provision of information to accompany the sale of treatments [8]. Available treatments may be clinically inappropriate and/or administered in doses that are outside of the therapeutic range [8]. Private sector providers may also have little financial incentive to distribute first-line anti-malarials for treatment and will instead sell a wide variety of low-cost anti-malarials [9, 10]. Similarly, while malaria rapid diagnostic tests (RDT) can accurately diagnose malaria and prevent the unnecessary use of artemisinin-based combination therapy (ACT), private sector providers may be reluctant to provide a confirmatory test given financial disincentives and a desire to profit through the sale of anti-malarial products [11, 12]. Moreover, providers, especially in the informal or unregulated sector, often have less training, including training on medicines that are not in the NTGs, which are subject to frequent changes given evolving drug resistance in the region [13]. These documented challenges with the private sector readiness and performance for malaria case management threaten recent elimination goals and strategies. To meet these elimination goals, it is imperative that the private sector is in alignment with national regulations, guidelines, and quality-assurance standards for malaria case management.

Given the role and diversity of the private sector across different countries and challenges with its performance, several efforts have been made to support and engage private sector providers to ensure high quality care or to prohibit this sector entirely from providing malaria case management services. This has included a PPM programme in Cambodia (since 2011) and the Lao PDR (since 2008) to regulate and license private for-profit facilities and pharmacies and to provide subsidized malaria commodities, training, and supervision. In Myanmar, private sector strengthening through the Artemisinin Monotherapy Replacement (AMTR) Project has been in place since 2012 to increase access to subsidized first-line treatments, including supportive interventions targeted at the informal private sector. In Thailand, the government banned the sale of anti-malarials in the private sector in 1995 to control the spread of drug resistant parasites. To date, the comparative performance of the different private sector anti-malarial markets across these countries has yet to be investigated.

Given the popularity of private sector providers for health services, the private sector can be an asset for accelerating progress towards national malaria elimination goals. However, private providers operating outside of regulation, national guidelines, and quality-assurance standards pose a serious threat to elimination goals [14]. Market landscaping is essential in an elimination setting [15] as it provides insights into the breadth and quality of private sector diagnosis, treatment, and reporting and identifies gaps and challenges in each country context. Information provided through a market landscape can help prioritize the specific outlets to target and identify the extent to which outlets are operating in accordance with the current regulatory environment. Evidence can be adapted to create strategies for engagement with the private sector within each country.

The aim of this paper is to review multi-country evidence of private sector adherence to national regulations, guidelines, and quality-assurance standards for malaria case management and to document current coverage of private sector engagement and support. This information can be used to target appropriate strategies designed to ensure private provider alignment with and contributions to national malaria control and elimination goals.

## Methods

ACTwatch was launched in 2008 by Population Services International (PSI) with support from the Bill and Melinda Gates Foundation. Details about the ACTwatch project and methodology have been published elsewhere [16, 17]. The goal of the project is to generate timely, relevant, and high quality evidence about anti-malarial and diagnostic markets for policy makers, donors, and implementing organizations. As of 2016, ACTwatch had gathered data from a total of 12 malaria endemic countries in sub-Saharan Africa and the GMS. This paper presents data from outlet surveys in four GMS countries collected in 2015 and 2016.

### Design and sampling

The ACTwatch outlet surveys were nationally-representative or sub-national surveys conducted among a sample of outlets stocking anti-malarial medicines and diagnostics. All categories of outlets with the potential to stock anti-malarials in both the public and private sectors were included in the study. In the public sector, this included government health facilities (hospitals, centres, clinics, and posts) and community health workers (CHW). Outlets sampled in the private sector included private for-profit health facilities (hospitals, centres, and clinics), pharmacies, drug stores, general retailers, and itinerant drug vendors (mobile vendors without a fixed service delivery point). Drug stores were not present in Myanmar and thus not represented as an outlet category. In Myanmar, permission was not received to include public health facilities, so these were excluded from the study.

Probability proportional to size (PPS) sampling was used to select administrative units for the surveys using each country’s national population sampling frames. Administrative units were clusters that typically had a population size of 10,000–15,000 inhabitants. As lists of all potentially eligible outlets were not routinely available, an outlet census was used to identify outlets for inclusion in the survey. To identify outlets, interviewers would walk systematically through each of the selected clusters looking for relevant outlets. Lists of registered outlets, such as public health facilities or pharmacies, were obtained prior to the data collection and used to help identify outlets. Local maps were also used to identify the catchment area of each selected cluster within a country. To identify itinerant drug vendors, congregation points or locations were identified using key informant interviews. These providers were approached by interviewers and asked if they had already participated in the survey to avoid duplication. Within each selected cluster, all outlet types with the potential to provide anti-malarials to consumers were screened. Outlets were eligible for a provider interview and malaria product audit if they met at least one of three study criteria: (1) one or more anti-malarials reportedly in stock on the day of the survey; (2) one or more anti-malarials reportedly in stock within the 3 months preceding the survey; and/or (3) malaria RDT in stock or malaria microscopy available on the day of the survey. The sampling strategy and stratification is summarized in Table 3.

**Table 3:** Summary of sampling procedures across the study countries

	Cambodia	Lao PDR	Myanmar	Thailand
Dates of data collection	August 17th to October 1st, 2015	November 18th to December 29th, 2015	August 18th, 2015, to January 4th, 2016	February 9th to March 22nd, 2016
Stratification	*National,* two domains based on the WHO evidence about artemisinin resistance to define a 3-tier stratification [45]: Tier 1 provinces (prioritized for immediate multifaceted response to contain or eliminate resistance) Tier 2 provinces (prioritized for intensified malaria control to reduce transmission and/or limit the risk of emergence or spread of resistant parasites)	*Sub*-*national,* five southern provinces	*National,* four geographic domains: Eastern domain Central domain Western domain Coastal domain	*Sub*-*national,* two geographic domains: Thai–Cambodia border areas Thai–Myanmar border areas
Number of clusters	160 Communes	77 Village groups	808 Wards and village tracts	194 Sub-districts

In the Lao PDR and Thailand, boundaries for the outlet census were extended to higher administrative units to cover a larger area for key outlets or areas. In the Lao PDR, this included oversampling of pharmacies and private for-profit health facilities at the district level. In Thailand, the geographic area for sampling outlets was extended to the district level for districts with an international border. This booster sampling strategy was used to expand the census and screening of pharmacies.

Myanmar had four geopolitical zones that were used as research domains. Since 2012, yearly sub-national surveys had been conducted in the Central and Eastern parts of the country as a means to monitor the AMTR project. The Eastern part of the country had been previously described as the AMTR project intervention area, given that several supportive interventions have been implemented in this part of the country [3, 18]. The Central domain had been described as the ‘Comparison’ area to observed differences between this area and the Eastern domain, where AMTR activities had been in place. Cambodia and Thailand had two research domains, and the study was stratified to deliver estimates for relevant research domains. Both Thailand and the Lao PDR were sub-national surveys, while Cambodia and Myanmar were nationally representative.

The study was designed to generate estimates for key market indicators within each domain. Minimum sample size requirements were calculated to estimate, with ±10% precision, the following indicators: (1) the proportion of private sector outlets with ACT medicine available, among outlets with anti-malarial(s) in stock on the day of the survey; and (2) proportion of outlets with malaria blood testing (RDT or microscopy) available, among outlets with anti-malarial(s) in stock on the day of the survey or within the past 3 months. The number of study clusters was calculated for each research domain based on the required number of anti-malarial stocking outlets and assumptions about the number of anti-malarial stocking outlets per cluster. Sample size requirements for follow-up surveys were calculated using information from previous survey rounds where available.

Data collection periods varied by country and over time but were typically implemented during the peak malaria transmission season for each country and lasted approximately 6 weeks, with the exception of Myanmar which took over 4 months.

### Training and data collection

Interviewer training consisted of standardized classroom presentations and exercises as well as a field exercise. Additional training was provided for supervisors and quality-controllers focused on field monitoring, verification visits, and census procedures. Data collection teams were provided with a list of selected clusters and official maps that illustrated administrative boundaries. In each selected cluster, fieldworkers conducted a full enumeration of all outlets that had the potential to provide anti-malarials. This included enumeration of outlets with a physical location, as well as identification of CHW and itinerant drug vendors using local informants.

Quality control measures implemented during data collection included questionnaire review by supervisors and interview verification visits conducted by quality controllers to between 10 and 20% of all outlets. Up to three visits were made to all outlets to complete the screening process, audit, and provider interview as needed.

As previously mentioned, a series of screening questions were administered at all outlets to determine eligibility for the survey. Following informed consent procedures, an audit of all available anti-malarial medicines and RDT was conducted. In addition to the product audit, a series of questions were administered to the senior-most provider regarding malaria case management knowledge and practices. Questions were also administered to providers to measure the extent to which they reportedly received supervision, training on NTG or malaria diagnostics, access to subsidized anti-malarials, and caseload reporting. Questions regarding access to subsidized anti-malarials and RDT were not administered in Myanmar.

All surveys were paper-based with the exception of Cambodia, where data were collected using Android phones and forms created using DroidDB (© SYWARE, Inc., Cambridge, MA, USA). Interviews were conducted in the local language using questionnaires that were translated from English to the local language and back to English to confirm translations.

### Data analysis

Double data entry was conducted using Microsoft Access (Microsoft Corporation, Redmond, WA, USA) with built-in range and consistency checks. Data were analysed across survey rounds using Stata (StataCorp College Station, TX). Sampling weights were calculated as the inverse of the probability of cluster selection. All point estimates were weighted using survey settings and all standard errors calculated taking account of the clustered and stratified sampling strategy.

Standard indicators were constructed according to definitions applied across the ACTwatch project and have been described in detail elsewhere [17, 19]. Briefly, anti-malarials identified during the outlet drug audit were classified as treatments found in the NTGs or not, and within the NTGs as first-line or second-line treatment for *P. falciparum* and *P. vivax* malaria. Drug audit information used for the classification included active ingredients, formulation, and strengths. Official NTGs in use at the time of the survey were used for the classification. Availability of NTG treatments at the outlet level was defined as availability of any component of what may be a multi-drug regimen. However, when one of the drugs was not an anti-malarial (e.g. antibiotics), the anti-malarial was only classified as a medicine in the NTGs if the partner antibiotic was available as well. The rationale for this classification is that if the anti-malarial medicine was present without the antibiotic, the anti-malarial could not be administered according to the NTGs. First-line and second-line treatment availability, and treatments not in the NTGs, were restricted to those outlets that had anti-malarials in stock.

RDT were classified as quality-assured or non quality-assured. Quality-assured RDT were RDT that were in compliance with the Global Fund Quality Assurance Policy on the Global Fund list of approved RDT products for procurement. The product catalogue number (PCN) was used to identify products on the Global Fund list of approved products. Availability of quality-assured RDT and non quality-assured RDT was restricted to outlets with an RDT in stock.

Anti-malarial market composition was defined as the proportion of outlets of each type, among outlets with anti-malarials in stock on the day of the survey. Market share, or the relative distribution of anti-malarials to individual consumers recorded in the drug audit, was standardized to allow meaningful comparisons between anti-malarials with different treatment courses and different formulations. The adult equivalent treatment dose (AETD) was defined as the amount of active ingredient required to treat an adult weighing 60 kg according to WHO treatment guidelines [2]. Provider reports on the amount of the drug sold or distributed during the week preceding the survey were used to calculate volumes according to type of anti-malarial. The volume of each drug was calculated as the number of AETDs that were reported to have been sold/distributed during the week preceding the survey. Measures of volume included all dosage forms to provide a complete assessment of anti-malarial market share.

## Results

In total, 76,168 outlets were screened for availability of anti-malarials and malaria diagnostics during the 2015 and 2016 outlet surveys: Cambodia (N = 26,664), the Lao PDR (N = 7586), Myanmar (N = 28,267) and Thailand (N = 13,651). For all surveys, the majority of outlets screened and with completed interviews were private sector outlets. Approximately 6500 full interviews were conducted (Cambodia, N = 1303; the Lao PDR, N = 724; Myanmar, N = 4395; and Thailand, N = 74) where a total of 11,437 anti-malarials and 4043 RDT were audited. Refer to Table 4 for a full breakdown of the screening and audit results for each country by sector.

**Table 4:** Results of the outlet census and AM/RDT product audit (N)

	Cambodia		Lao PDR		Myanmar		Thailand	
	Public	Private	Public	Private	Public^b^	Private	Public	Private
Screened	604	26,060	558	7028	2737	25,530	355	13,296
Eligible^a^	558	750	258	467	1449	2948	79	25
Interviewed	557	746	258	466	1463	2932	49	25
Anti-malarial(s) in stock	467	391	236	394	1263	2596	72	19
Anti-malarial(s) out of stock, in stock in the past 3 months	75	179	19	30	119	294	0	1
Malaria testing available	15	176	3	42	81	42	7	5
Anti-malarials audited	759	531	773	217	4227	4642	247	41
Malaria RDT audited	605	592	893	266	1188	447	44	8

^a^Outlets are eligible for the outlet survey if they either (1) have anti-malarials currently in stock at the time of the interview, (2) have stocked anti-malarials in the past 3 months, or (3) currently stock malaria microscopy or malaria RDT

^b^In Myanmar, public health facilities were not included in the study. The numbers in this table reflect the results of the outlet census and audit of CHW in Myanmar

Across the facility types, availability of at least one anti-malarial among all screened outlets varied. Anti-malarials were commonly available in public health facilities in Cambodia (77.9%), the Lao PDR (97.8%) and in Thailand (94.9%). Private sector availability was lower, and most common among private for-profit facilities in Cambodia (31.0%), the Lao PDR (36.2%) and Myanmar (50.4%). In the Lao PDR, 70.6% of pharmacies had at least one anti-malarial in stock. Across other private sector outlet types, anti-malarials were less commonly available (<20%) (Additional file 1).

### Market composition

**Fig. 1:**
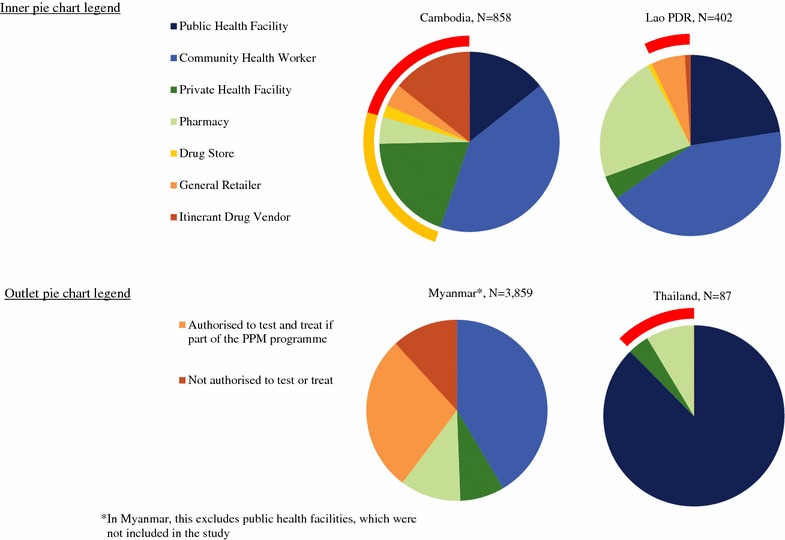
Anti-malarial market composition

Figure 1 shows that, in terms of absolute number of places where anti-malarial medicines were available, there was considerable diversity in the types of outlets providing malaria treatment across countries. In Cambodia, the private sector market composition was comprised primarily of private for-profit facilities and itinerant drug vendors. In the Lao PDR and Thailand, the private sector service delivery points were typically pharmacies. In Myanmar, the private sector composition comprised primarily of general retailers, but itinerant drug vendors and pharmacies were also common. In the public sector, across Cambodia, the Lao PDR, and Myanmar, CHW composed just over 40% of the market composition, and findings were similar between these three countries. In Thailand, public health facilities were the most common type of outlet stocking anti-malarials (87.6%), but in the Lao PDR and Cambodia, public health facilities were less than 25% of the anti-malarial service delivery points.

The outer pie chart in Fig. 1 illustrates outlets that are authorized to test for and treat malaria, according to national policy. All of the private sector outlets in Thailand were not authorized to test for or treat malaria. In Cambodia, half of the private sector anti-malarial market composition consisted of outlets that were not authorized to test for or treat malaria, and in the Lao PDR this included one in four private sector anti-malarial stocking outlets. In contrast, all private sector outlets in Myanmar were authorized to test for and treat malaria.

### Anti-malarial availability

**Fig. 2:**
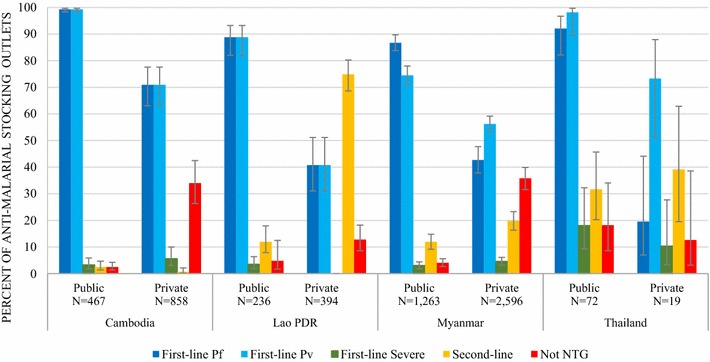
Anti-malarial availability in the public and private sectors

Availability of first-line, second-line, and treatment not indicated in the NTGs among anti-malarial stocking outlets is shown in Fig. 2. Treatment categories for each country are defined in Table 2. Availability of NTG treatments at the outlet level were defined as availability of any component of what may be a multi-drug regimen according to each country’s recommended guidelines.

#### Public sector

Among outlets stocking at least one anti-malarial in the public sector, availability of any component of the first-line treatment for uncomplicated *P. falciparum* or *P. vivax* malaria was greater than 85% and highest in Cambodia, where there was almost universal coverage (99%). One exception to this was found among CHW in Myanmar, where slightly less than three-quarters of these outlets had any component of the first-line treatment for *P. vivax* available (74.5%). Availability of any component of the first-line treatment for severe malaria was less than 5% of the anti-malarial stocking public sector in Cambodia, the Lao PDR, and Myanmar. Stocking rates of the first-line treatment for severe malaria were slightly higher in Thailand’s public sector (18.1%).

In the anti-malarial stocking public sector, availability of any component of the second-line treatment was low in Cambodia (2.5%), the Lao PDR (12%), and Myanmar (12%). In Thailand, around one in three public sector outlets were stocking any component of the second-line treatment (31.7%). Availability of treatment not in the NTGs was generally less than 5% across Cambodia, the Lao PDR, and Myanmar’s public sector. In Thailand, 18.2% of the anti-malarial stocking public sector had treatments that were not in the NTGs.

#### Private sector

Among outlets stocking at least one anti-malarial in the private sector, availability of any component of the first-line treatment for uncomplicated *P. falciparum* or *P. vivax* malaria was variable across countries and lower than the public sector. In Cambodia, availability of the first-line treatment for *P. falciparum* or *P. vivax* (ACT) was 70.9%, and in the Lao PDR, less than half of the anti-malarial stocking outlets had the first-line *P. falciparum* or *P. vivax* treatment (ACT) in stock (40.8%). In Myanmar and Thailand, less than half of the private sector had any component of *P. falciparum* treatment (ACT and/or primaquine) (42.7 and 19.6% respectively). Availability of any component of *P. vivax* treatment (chloroquine and/or primaquine) was higher in these countries (56.2 and 73.3%, respectively). Availability of any component of the first-line treatment for severe malaria was generally less than 5% of the anti-malarial stocking private sector across all countries but was slightly higher in Thailand (10.3%).

In the private sector, across all countries, availability of any component of the second-line treatment among anti-malarial stocking outlets was variable and highest in the Lao PDR (74.9%) followed by Thailand (39.1%). In these countries, availability of second-line treatment was greater than availability of the first-line treatment for uncomplicated malaria. In Myanmar, second-line treatment was available in one in five anti-malarial stocking private sector outlets (19.8%) and rarely present in Cambodia (0.7%). The types of second-line treatment medicines that were available were different across countries. In the Lao PDR, this was predominately second-line treatment for *P. vivax* malaria (chloroquine tablets, branded as Maraquin^®^) and Maraquin was included in the national list of registered medicines. In Myanmar, this was second-line treatment for severe malaria (quinine and artemether liquid injections), and in Thailand, this was second-line treatment for *P. falciparum* malaria (quinine + doxycycline tablets).

Approximately one in three private sector outlets in Cambodia (34.0%) and Myanmar (35.8%) stocked medicines that were not included in the NTGs. In Cambodia, these medicines were most commonly chloroquine tablets, artemisinin piperaquine tablets, and non-FDC artesunate mefloquine tablets. In Myanmar, these medicines were commonly oral artemisinin monotherapy. See Additional file 2 for a comprehensive list of all audited anti-malarials that were not included in the NTGs.

#### Types of private sector outlets stocking non-first-line treatments

Table 5 illustrates the availability of any component of the second-line treatment and treatment not included in the NTGs among anti-malarial stocking private sector outlet types. Availability of second-line treatment was most common among pharmacies (the Lao PDR, 72.4%; Myanmar, 27.2%; and Thailand, 28.1%) and itinerant drug vendors (the Lao PDR, 57.6%; Myanmar, 46.9%). Private for-profit facilities were also found to commonly stock any component of the second-line treatment in some countries (the Lao PDR, 49.3%; Thailand, 70.0%). Availability of private sector second-line treatment was observed across all outlets in the Lao PDR and was rarely found in Cambodia’s private sector.

**Table 5:** Percentage of anti-malarial stocking private sector outlets with non-first-line anti-malarials available

	Private for-profit health facility % (CI)	Pharmacy % (CI)	Drug store % (CI)	General retailer % (CI)	Itinerant drug vendor % (CI)	Private sector total % (CI)
Cambodia	N = 186	N = 45	N = 22	N = 29	N = 109	N = 391
Second-line	0.3	0.0	6.8	0.0	0.6	0.7
	(0.1, 2.0)	–	(1.9, 21.4)	–	(0.1, 3.7)	(0.2, 2.2)
Not in NTGs	12.8	16.3	45.3	100.0	48.4	34.0
	(7.8, 20.4)	(9.0, 27.7)	(26.1, 66.1)	–	(38.2, 58.6)	(26.4, 42.5)
Lao PDR	N = 56	N = 309	N = 3	N = 23	N = 3	N = 394
Second-line	49.3	72.4	100.0	96.9	57.6	74.9
	(33.7, 65.0)	(64.8, 78.9)	–	(83.0, 99.5)	(40.9, 72.8)	(68.6, 80.3)
Not in NTGs	11.1	15.3	0.0	0.0	57.6	12.8
	(5.4, 21.5)	(10.4, 21.9)	–	–	(40.9, 72.8)	(8.9, 18.2)
Myanmar	N = 314	N = 522	N/A^a^	N = 1341	N = 419	N = 2596
Second-line	15.6	27.2	–	6.2	46.9	19.8
	(9.2, 21.9)	(20.8, 33.5)		(3.4, 9.0)	(40.7, 53.1)	(16.3, 23.3)
Not in NTGs	20.3	43.0	–	43.0	29.5	35.8
	(13.8, 26.8)	(35.6, 50.4)		(36.7, 49.3)	(23.0, 36.0)	(31.6, 39.9)
Thailand	N = 9	N = 10	N = 0	N = 0	N = 0	N = 19
Second-line	70.0	28.1	–	–	–	39.1
	(23.8, 94.6)	(10.5, 56.7)	–	–	–	(19.6, 62.9)
Not in NTGs	48.1	0.0	–	–	–	12.6
	(13.7, 84.4)	–	–	–	–	(3.2, 38.6)

^a^ Drug stores do not exist in Myanmar

Availability of treatment not in the NTGs was most common among itinerant drug vendors (Cambodia, 48.4%; the Lao PDR, 57.6%; Myanmar, 29.5%) and general retailers (Cambodia, 100%; Myanmar, 43.0%). Availability of treatment not in the NTGs was common (>20%) across all private sector outlet types in Myanmar.

### Anti-malarial market share

**Fig. 3:**
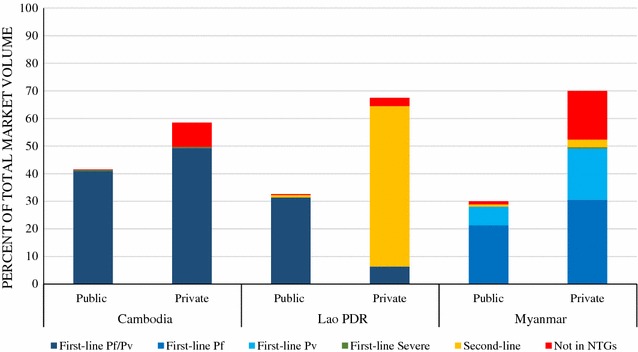
Anti-malarial market share in the public and private sectors

Figure 3 shows the market share of different categories of anti-malarials sold or distributed in the 7 days prior to the survey. The private sector played a larger role than the public sector in the distribution of anti-malarials. The majority of anti-malarials distributed in Cambodia and Myanmar were first-line *P. falciparum* or *P. vivax* treatments (90.3 and 77.1% respectively). In the Lao PDR, only 37% of the anti-malarial market share was first-line treatment for *P. falciparum* or *P. vivax* malaria. In all three countries, public sector market share was dominated by first-line *P. falciparum* or *P. vivax* treatment. In the private sector, the anti-malarials distributed included second-line treatment and treatment not in the NTGs. In the private sector in Cambodia and Myanmar, 8.8 and 17.6% of national anti-malarial market share, respectively, were treatment not in the NTGs. In the Lao PDR, 59.0% of national market share was private sector second-line treatment. Approximately 9 in 10 anti-malarials distributed in the Lao PDR’s private sector were second-line treatments.

### Private-sector anti-malarials not included in national treatment guidelines

Additional file 2 includes a list of all audited anti-malarials that were not indicated in NTGs for each of the study countries. Product characteristics such as generic name, formulation, brand name, manufacturer, country of manufacturer, registration status, number of products audited, and outlet type are described. There were 9 unique products audited in Cambodia, 4 in the Lao PDR, 26 in Myanmar, and 3 in Thailand. All anti-malarials except for 2 (Mephaquin^®^ manufactured by Mepha in Switzerland and Malanil^®^ manufactured by Glaxosmithkline in Canada) were manufactured in Asian countries (China, India, the Lao PDR, Myanmar, Pakistan, Thailand, Vietnam).

In Cambodia, all audited anti-malarials that were not indicated in the NTGs were either artemisinin piperaquine tablets (n = 31), artesunate tablets (n = 1), chloroquine tablets (n = 67), or non-FDC artesunate mefloquine tablets (n = 27). No audited products in Cambodia were manufactured locally, and only 1 was included in the national list of registered medicines (chloroquine tablets manufactured by Acdhon). In the Lao PDR, the majority of products audited that were not in the Lao PDR NTGs included chloroquine injections (n = 45) and syrups (n = 4). The chloroquine injection audited was Malacin^®^ and was included in the national list of registered medicines, while the chloroquine syrup was branded Chloquine^®^ and, although manufactured locally in the Lao PDR, was not included in the list of registered medicines. In Myanmar, most products audited were artemether (n = 57), artesunate (n = 891), and SP tablets (n = 66). Of the artesunate tablets audited, 88% (n = 784) were manufactured by Mediplantex^®^ in Vietnam. Several products (unbranded artesunate and mefloquine tablets, and SP Pyrixine^®^) were manufactured locally by Myanmar/Tatmadaw Pharmaceutical Factory. None of these aforementioned products found in Myanmar were included in the national list of registered medicines. There were only 3 products audited in Thailand that were not included in the NTGs.

### Malaria confirmatory testing availability and types of RDT products

**Fig. 4:**
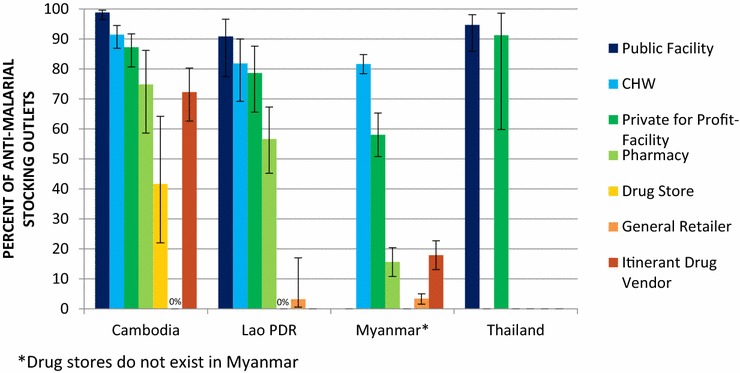
Availability of any confirmatory test in the public and private sectors

Availability of any test among the anti-malarial stocking public health facilities was greater than 90% across Cambodia (98.8%), the Lao PDR (90.8%) and Thailand (94.7%). Availability among anti-malarial stocking CHW was greater than 80% in Cambodia (91.4%), the Lao PDR (81.8%) and Myanmar (81.6%). The private sector ranged from 87.2% of anti-malarial stocking private for-profit facilities in Cambodia, 78.6% in the Lao PDR, 58.0% in Myanmar and 91.2% in Thailand. Among anti-malarial stocking pharmacies, availability ranged from 74.8% in Cambodia, 56.6% in the Lao PDR, 15.6% in Myanmar. Availability was 17.9% among anti-malarial stocking itinerant drug vendors in Myanmar and less than 5% of general retailers in the Lao PDR and Myanmar (Fig. 4).

**Fig. 5:**
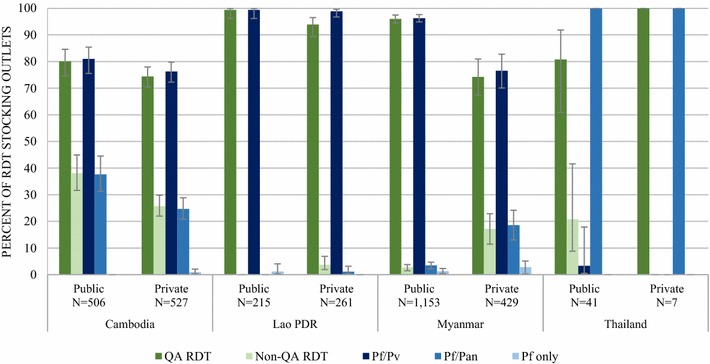
Availability of RDT in the public and private sectors​ with and without Quality Assurance status and according to parasite species detection

Among outlets stocking any RDT, availability of malaria RDT with and without Global Fund Quality Assurance status and according to parasite species detection among RDT-stocking outlets is shown in Fig. 5. Amongst the RDT-stocking public sector, availability of quality-assured RDT ranged from 99.3% in the Lao PDR to 80.1% in Cambodia. Public sector availability of non quality-assured RDT was 38.1% in Cambodia, 20.8% in Thailand, and was negligible or non-existent across the other countries.

Amongst the RDT-stocking private sector, 100% of private facilities in Thailand had quality-assured RDT in stock and almost all private facilities in the Lao PDR (94%). In Cambodia and Myanmar, 3 in 4 private sector RDT-stocking facilities had quality-assured RDT available. Private sector availability of non quality-assured RDT was observed in 25.7% of facilities in Cambodia and 17.2% in Myanmar. Private sector availability of non quality-assured RDT was negligible or non-existent in the Lao PDR and Thailand.

Almost all RDT audited in the four countries could detect *P. falciparum* and either *P. vivax* (*Pf/Pv*) or other species (*Pf*/*Pan*). Approximately three-quarters of RDT-stocking outlets in Cambodia stocked *Pf*/*Pv* RDT (public 81.0%, private 76.3%) and one-quarter stocked *Pf*/*Pan* (public 37.7%, private 24.7%). Almost all RDT-stocking outlets in the Lao PDR stocked *Pf*/*Pv* RDT (public 99.4%, private 98.9%). Similarly, outlets stocking RDT in Myanmar’s public sector almost exclusively stocked RDT that could detect *Pf*/*Pv* (96.3%), while 76.5% of private sector outlets stocked *Pf*/*Pv* RDT and 18.6% stocked *Pf/Pan*. All public and private RDT-stocking outlets in Thailand stocked *Pf*/*Pan* RDT.

### Anti-malarial market share: volumes distributed in outlets with and without confirmatory testing

**Fig. 6:**
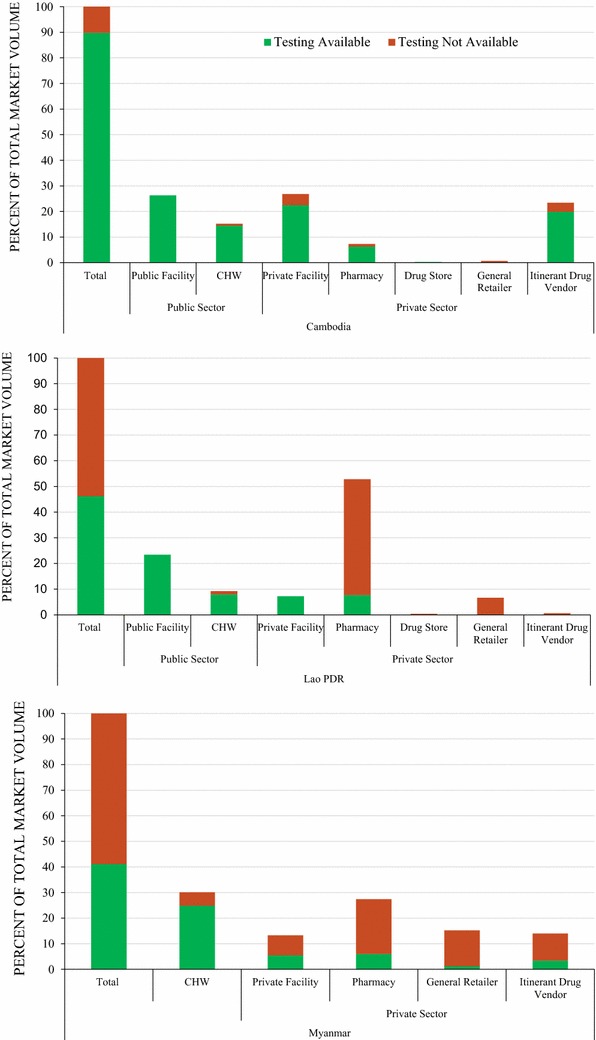
Anti-malarial market share: volumes distributed in outlets with and without confirmatory testing

In Cambodia, 90% of all anti-malarials distributed were distributed by outlets that had confirmatory testing available (Fig. 6). This includes all anti-malarials distributed by public health facilities and most anti-malarials distributed by CHWs. Over half of all anti-malarial distribution was by outlets that did not have confirmatory testing available in the Lao PDR (54%) and Myanmar (59%). Anti-malarial distribution by outlets without confirmatory testing available occurred primarily in pharmacies in the Lao PDR, where 45.1% of the total markets share was distributed through outlets without testing. In Myanmar, distribution of anti-malarials without  confirmatory testing available was common across all private sector outlet types.

### Private sector support and engagement

In all three countries with an authorized private sector, private health facilities and pharmacies more commonly received some form of support compared to other private sector outlet types, but some country differences were observed (Table 6). With regards to training and supervision, in Cambodia and the Lao PDR, over 20% reportedly received training on malaria diagnosis (Cambodia, 23.9%; the Lao PDR, 31.7%) or the NTGs (Cambodia, 22.2%; the Lao PDR, 22.0%). Less than 10% of providers in Myanmar reportedly received either training. Close to half of the providers in the Lao PDR (46.7%) reportedly received some form of supervisory or regulatory visit in the past 12 months. This was lower in Myanmar and Cambodia (19.9 and 10.6%, respectively). In Myanmar, this supervision was most common among private for-profit facilities and general retailers.

**Table 6:** Percent of private sector providers that reportedly received malaria case management training within the last year, supervision within the last year, subsidized anti-malarials and/or malaria RDT, and report caseload data

	Private for- profit health facility % (CI)	Pharmacy % (CI)	Drug store % (CI)	General retailer % (CI)	Itinerant drug vendor % (CI)	Private sector total % (CI)
Cambodia	N = 318	N = 99	N = 46	N = 39	N = 235	N = 737
Training on malaria diagnosis	36.6	43.3	16.4	3.2	9.3	23.9
	(29.3, 44.6)	(31.9, 55.4)	(7.8, 31.2)	(0.7, 13.8)	(6.3, 13.7)	(19.5, 28.9)
Training on NTGs	34.8	41.5	15.2	3.2	7.3	22.2
	(27.1, 43.3)	(31.0, 52.7)	(6.8, 30.5)	(0.7, 13.8)	(4.7, 11.3)	(17.8, 27.4)
Supervision or regulatory visit	18.4	14.7	9.4	0.0	2.9	10.6
	(13.8, 24.1)	(7.6, 26.5)	(2.5, 29.8)	–	(1.4, 5.8)	(7.5, 14.7)
Subsidized anti-malarials	37.9	52.9	12.2	0.0	14.3	26.9
	(32.1, 44.1)	(43.2, 62.4)	(6.1, 22.9)	–	(10.3, 19.5)	(23.0, 31.1)
Subsidized RDT	40.1	52.1	27.8	0.0	15.0	29.0
	(34.2, 46.2)	(43.9, 60.2)	(15.9, 44.1)	–	(10.6, 20.9)	(25.0, 33.3)
Report caseload data	32.4	17.2	8.2	0.0	5.9	17.5
	(24.2, 41.9)	(9.5, 29.3)	(1.8, 30.1)	–	(3.0, 11.5)	(12.1, 24.5)
Lao PDR	N = 94	N = 330	N = 6	N = 30	N = 5	N = 465
Training on malaria diagnosis	36.6	42.8	4.6	3.7	21.0	31.7
	(25.5, 49.3)	(33.1, 53.1)	(0.6, 26.1)	(0.6, 19.5)	(2.9, 70.3)	(23.9, 40.7)
Training on NTGs	23.3	27.7	30.3	3.7	21.0	22.0
	(16.4, 31.9)	(21.0, 35.5)	(6.1, 74.5)	(0.6, 19.5)	(2.9, 70.3)	(16.7, 28.4)
Supervision or regulatory visit	50.2	62.1	16.7	11.5	0.0	46.7
	(37.9, 62.5)	(50.4, 72.6)	(3.7, 51.0)	(5.7, 21.9)	–	(37.7, 56.0)
Subsidized anti-malarials	41.8	50.4	0.0	2.5	0.0	36.2
	(28.8, 56.1)	(39.7, 61.0)	–	(0.4, 14.5)	–	(27.9, 45.3)
Subsidized RDT	40.4	52.0	25.7	2.5	0.0	37.9
	(26.7, 55.9)	(40.7, 63.0)	(4.1, 73.5)	(0.4, 14.5)	–	(29.2, 47.4)
Report caseload data	65.1	54.5	0.0	2.5	0.0	41.9
	(53.4, 75.2)	(42.7, 65.9)	–	(0.4, 14.5)	–	(33.2, 51.1)
Myanmar^a^	N = 354	N = 576	N/A^b^	N = 1487	N = 508	N = 2925
Training on malaria diagnosis	22.6	12.1	–	3.2	4.4	8.0
	(17.4, 27.7)	(7.1, 17.0)		(1.4, 5.0)	(2.3, 6.4)	(5.9, 10.2)
Training on NTGs	23.6	9.3	–	6.4	6.7	9.4
	(18.1, 29.1)	(4.9, 13.7)		(3.6, 9.3)	(3.4, 9.9)	(6.9, 11.8)
Supervision or regulatory visit	32.0	16.7	–	19.2	12.5	19.9
	(25.5, 38.6)	(12.7, 20.8)		(16.0, 22.3)	(8.6, 16.3)	(17.1, 22.7)
Report caseload data	40.3	4.7	–	2.4	7.7	9.4
	(31.0, 49.6)	(2.6, 6.9)		(1.0, 3.8)	(4.3, 11.1)	(6.7, 12.1)

Providers in outlets that have malaria testing and/or treatment available on the day of the survey or in the past 3 months

^a^ Questions about subsidized anti-malarials and RDT were not asked in Myanmar

^b^ Drug stores do not exist in Myanmar

In terms of access to subsidized commodities, almost 40% of providers in the Lao PDR reported having received subsidized or free anti-malarials or RDT (anti-malarials, 36.2%; RDT, 37.9%). In Cambodia, around 1 in 4 providers reportedly received subsidized anti-malarials (26.9%) and this was most commonly reported by private for-profit facilities (40.1%) and pharmacies (52.1%) but also among itinerant drug vendors (15.0%).

Caseload data reporting was highest in the Lao PDR (41.9%), common among private for-profit facilities (65.1%) and pharmacies (55.4%) and negligible or zero among other outlet types. In Cambodia’s private sector, 17.5% of facilities reportedly report caseload data, and while most common among private for-profit facilities (32.4%), also included pharmacies (17.2%), drug stores (8.2%), and itinerant drug vendors (5.9%). In Myanmar, private sector caseload reporting was reported among fewer than 10% of outlets and was most common among private for-profit facilities (40.3%).

## Discussion

Findings from this multi-country study suggest that the private sector for malaria case management in Cambodia, Myanmar, and the Lao PDR is generally in alignment with national regulations, treatment guidelines and quality-assurance standards. However, important gaps persist and pose a threat to national malaria control and elimination goals.

### Anti-malarial availability among informal and unauthorized private-sector outlets

In 1995, Thailand banned the sale of anti-malarials in the private sector as a method of controlling the spread of drug resistant parasites. Of the 13,000 private-sector outlets screened during the Thailand survey, only 19 were found to be stocking anti-malarials. These results suggest that the long-standing private-sector ban on anti-malarial sales in Thailand has been widely enforced.

The private-sector market composition was substantial in Cambodia, the Lao PDR, and Myanmar, where one-third or more of anti-malarial service delivery points were found in the private sector. Unlike Myanmar, where all private-sector outlet types were authorized to test for and treat malaria, in Cambodia and the Lao PDR, drug stores, general retailers, and itinerant drug vendors were required to refer patients with fever to public-sector outlets, private health facilities, or pharmacies for appropriate care [20]. However, nearly half of the private-sector providers in Cambodia and approximately one-quarter in the Lao PDR were unauthorized drug stores, general retailers, or itinerant drug vendors, which typically lack formal ties to the public health system and national malaria surveillance systems [15]. Such outlets, therefore, pose a threat to case management according to NTGs.

Almost half of the private-sector outlets in Cambodia were unauthorized outlets, which is of importance given national efforts to increase regulation of the informal private sector as part of the country’s elimination plans. In 2010, the Cambodian government created a new police force exclusively to impose a ban on private anti-malarial drug sellers. Previous ACTwatch outlet survey data show a substantial decline in the relative number of anti-malarial stocking drug stores and general retailers since 2009, which has largely been attributed to increased regulation of the private sector [4]. However, the current survey indicates that there are still a substantial number of unauthorized outlets carrying anti-malarials. This may reflect continued patient demand for case management services across different outlet types as well as motivation among unauthorized providers to provide services to meet the demand. This may also reflect inadequate capacity of inspection and judiciary agencies, and a lack of resources to implement routine inspections as evidenced by other research [21, 22]. A review by Montagu and Goodman regarding the regulation of the private sector in developing countries has shown that regulatory approaches face persistent challenges [23]. Montagu and Goodman conclude that increased regulatory capacity should be the medium-term and long-term priority for developing countries and that short-term attention should be focused on interventions that encourage private providers to improve the quality and coverage of their care, thus allowing them to advance their own financial interests.

Approximately two-thirds of the unlicensed outlets in Cambodia were itinerant drug vendors. Although more research is needed to better understand the role itinerant drug vendors play, the current survey indicates that these providers were an important community-level anti-malarial access point [24]. In Cambodia there is evidence that itinerant drug vendors often have some sort of health qualification and have in the past been, or are currently affiliated with, public or private health facilities or pharmacies [25]. As such, it may be possible to formally engage these providers through private-sector mechanisms. One option would be to integrate these providers into the formal health system through training, supervision, business incentives, and accreditation [26]. Several malaria-endemic countries which have incorporated itinerant drug vendors into the private sector have documented improvements in provider knowledge and performance [27]. This option further speaks to the recommendations by Montagu and Goodman that would allow for the improvement of the quality and coverage of private sector care, while advancing providers’ own financial interests. The integration of itinerant drug vendors into the formal private sector could be operationalized through Cambodia’s PPM programme by incorporating these providers into the existing strategy. As the current PPM mechanism aims to train private providers on appropriate malaria diagnosis, treatment, and referral procedures, this would allow for a more regulated inclusion of the private sector in malaria case management while still adhering to national guidelines [28].

Myanmar represents a unique situation in that a large majority of the private sector comprises itinerant drug vendors and general retailers but in contrast to other GMS countries, these outlet types are permitted to test for and treat malaria. Private-sector strategies through the AMTR project in the Eastern part of the country have leveraged these providers to increase access to quality-assured, subsidized ACT medicines, and more recently RDTs [3, 29]. Other supportive strategies have included engaging with general retailers and itinerant drug vendors through product promoters and provider behaviour change communication, and several positive outcomes of these strategies have been documented [3]. However, while these providers have received access to subsidized commodities and supportive interventions, they are not currently part of a national strategy that actively registers, trains and supervises these outlets. Furthermore, most private-sector engagement has historically taken place in the Eastern part of the country. Given this, Myanmar’s findings do pose some challenges in the context of elimination strategies. In the absence of formally regulating these private-sector outlets, it will be challenging to routinely monitor and supervise providers, or obtain malaria surveillance data from them, which is a cornerstone of elimination strategies [15]. For example, data from Myanmar’s survey shows that only 3% of general retailers provide any sort of caseload data. The extent to which these types of outlets can be part of the broader elimination efforts, in the absence on national strategies to regulate, train and supervise these providers, needs to be determined. Initiatives from neighbouring countries in Cambodia and the Lao PDR, to formally incorporate such outlets into a programme similar to PPM will be useful to draw upon.

### Alignment of anti-malarial availability and distribution with national guidelines

According to NTGs, malaria cases confirmed with blood testing should be treated with first-line drugs. Nearly three-quarters of anti-malarial stocking private-sector outlets stocked *P. falciparum* and *P. vivax* first-line treatments for uncomplicated malaria in Cambodia and approximately half of private-sector outlets in the Lao PDR and Myanmar. In all countries, availability of *P. falciparum* and *P. vivax* first-line treatment was highest in private for-profit health facilities and pharmacies compared to availability in drug stores, general retailers, and itinerant drug vendors. The majority of anti-malarials distributed were *P. falciparum* and *P. vivax* first-line treatments in Cambodia (~85%) and Myanmar (~70%), however, the market share for first-line treatments in the private sector was very low in the Lao PDR (<10%). These results suggest that in Cambodia and Myanmar, most of the anti-malarial distribution was in accordance with NTGs, but there are notable gaps in the Lao PDR’s private sector.

In the GMS, both *P. falciparum* and *P. vivax* malaria account for a significant proportion of clinical cases [14]. Although first-line treatment for uncomplicated *P. falciparum* and *P. vivax* malaria is the same in some countries (e.g. Cambodia, the Lao PDR), second-line treatment and treatment for specific populations (e.g. pregnant women) differs, necessitating RDT with the capability to differentially diagnose *P. falciparum* from *P. vivax* malaria. Almost all RDT audited during the outlet surveys in Cambodia, the Lao PDR, Myanmar, and Thailand were *Pf/Pv* or *Pf/Pan* RDT. The small number of RDT audited in Cambodia, the Lao PDR, and Myanmar that could only detect *P. falciparum* malaria were manufactured by Standard Diagnostics (Bioline^®^) or Orchid Biomedical Systems (Paracheck^®^). These results suggest that where RDT testing is available and implemented, providers are able to identify parasite species to facilitate treatment according to treatment guidelines.

### Misalignment of anti-malarial availability and distribution with national guidelines

Within the GMS, adherence to first-line guidelines is essential. NTGs are regularly updated to reflect the latest evidence on which anti-malarials remain efficacious for parasite clearance [1]. This is perhaps most notable in Cambodia, which has seen several revisions to the first-line treatment over the past decade in response to drug resistant parasites, though recent changes to the NTGs have been observed across all the study countries. The continued availability and use of medicines that are not in the NTGs, or misuse or inappropriate use of second-line treatments in the private sector, not only threatens effective malaria control, but also national and regional elimination strategies and goals.

#### Private-sector second-line treatment

Second-line treatment should be utilized only after treatment failure with the first-line drug. Therefore, the availability of the second-line drug should be limited to public health facilities equipped to detect and manage first-line treatment failure. Second-line treatment is not expected to be available within private-sector outlets, especially pharmacies, drug stores, general retailers, and itinerant drug vendors that are not trained or authorized to manage treatment failure.

Second-line treatment availability in the private sector was high in the Lao PDR (~75%), low in Myanmar (~20%), and negligible in Cambodia (<1%). In Myanmar, second-line treatment accounted for 4% of private-sector market share, whereas in the Lao PDR, second-line treatment distribution dominated the market, accounting for close to 60% of overall market share and most of private-sector market share. Such high second-line treatment market share, despite moderate private sector availability of first-line *P. falciparum* and *P. vivax* treatment, suggests that factors other than availability are driving private-sector anti-malarial distribution in the Lao PDR.

Nearly all second-line treatment distributed in the Lao PDR was chloroquine, which is indicated for treatment of *P. vivax* malaria after AL treatment failure. Chloroquine should only be stocked at health facilities with the necessary equipment and skilled staff required to detect and manage treatment failure, however, in the Lao PDR, more than three-quarters of second-line treatment was distributed at pharmacies. This finding suggests that chloroquine was being used inappropriately to treat patients presenting for the first time with signs and symptoms of malaria. Furthermore, it is estimated that close to two-thirds of malaria cases in the Lao PDR are *P. falciparum* infections [14], which suggests chloroquine could also have been used to treat patients indiscriminately who may have *P. falciparum* malaria, for which chloroquine has been shown to have high treatment failure rates [30].

Regulation and removal of chloroquine from the Lao PDR private-sector outlets is urgently needed to facilitate sale and use of first-line *P. falciparum* and *P. vivax* treatment. Removing this product could be complex given that the majority of chloroquine distributed in the Lao PDR was Maraquine^®^ brand, manufactured locally by CBF Pharma, and included in the national list of registered medicines [25]. Removing a locally manufactured product from the market could have potential economic repercussions or provoke political sensitivities. As it is unlikely that local manufacturers will have the technical expertise, raw materials, quality standards, and production and laboratory equipment to produce the first-line ACT treatment [31] or to receive GMP status to enable purchase of the medicines using international donor funds, other compensation or incentive schemes may be necessary to halt the production of locally manufactured chloroquine. While some may argue that there are opportunities to work with local manufacturers to support the introduction of GMP and internal quality assurance in local pharmaceutical factories [32], others have concluded that investment to promote local manufacturing of medicines could be better used to promote health infrastructure [33]. Further attention is needed to actively engage with the manufacturer and advocate for halting the local distribution of this product.

#### Private-sector treatment that is not in the NTGs

The availability and distribution of treatments that are not in the NTGs should be carefully assessed, particularly in the context of elimination strategies and goals. Treatments that are not included in the NTGs, especially oral artemisinin monotherapy, not only pose a threat to patient health and safety and have the potential to delay parasite clearance and drive drug resistance [34, 35], but also pose a threat to effective malaria control and elimination goals. NTGs are regularly updated to reflect the latest evidence on which anti-malarials remain efficacious for parasite clearance, and thus it is of utmost importance that patients and providers adhere to these guidelines.

Availability and market share of treatment not in the NTGs differed by country and outlet type. In Cambodia and Myanmar, approximately 1 in 3 private-sector outlets stocked treatment not in the NTGs, accounting for 15 and 25% of private-sector market share, respectively. In Cambodia, a large majority of this treatment was chloroquine, while in Myanmar, treatment not in the NTGs was predominantly oral artemisinin monotherapy. The majority of these treatments in Cambodia and Myanmar were stocked by general retailers and itinerant drug vendors. Availability of treatment not in the NTGs was lower in the Lao PDR but still notable, with more than one in seven private-sector outlets stocking treatment that was not in the NTGs.

In Myanmar, 14 unique brands of oral artemisinin monotherapy were audited, primarily at general retailers, pharmacies, and itinerant drug vendors. Unbranded artesunate tablets manufactured by Mediplantex^®^ (Vietnam) accounted for nearly three-quarters of all anti-malarials not included in the NTGs. While several strategies have been in place to remove this medicine from the market, including the aforementioned AMTR project and a 2012 ban on oral artemisinin monotherapy, availability and market share was widespread. Reasons for the widespread availability and distribution may be in part attributed to an incomplete ban on oral AMT, which permits the continued importation of this medicine from manufacturers for up to 5 years from when the ban was first implemented. Several strategies are urgently needed to ensure the removal of this medicine, including enforcement of the ban. These could include active efforts to remove the product from the shelves and/or communications campaigns to promote provider compliance with the ban [4].

In Cambodia, the majority of audited anti-malarials that were not in the NTGs were either non-FDC ASMQ, artemisinin piperaquine, or chloroquine tablets, and of the nine specific brands audited, none were included in the country’s drug registry [24]. In 2009, chloroquine was the first-line *P. vivax* malaria treatment in Cambodia, but in 2011, first-line treatment changed to DHA PPQ or ASMQ after evidence of chloroquine treatment failure [36]. Audited chloroquine products included Nitaquin^®^ manufactured by Utopian and unbranded chloroquine manufactured by Acdhon, both of which were manufactured in Thailand. The availability of chloroquine points to the need for tighter regulation, registration of anti-malarials, and stricter importation laws, including tightened importation controls, to ensure these medicines are removed from the market. Measures may also include passing a law to ban chloroquine from the market, similar to the one passed for oral artemisinin monotherapy, which has been a successful in Cambodia [4].

The current PPM programmes implemented in Cambodia and the Lao PDR provide an opportunity and a strong foundation to scale up access to first-line treatments in the private sector and remove any unwanted medicines from the shelves. Similar accreditation programmes that combine training, business incentives, supervision, and regulatory enforcement have been successful at improving the quality of medicines and services provided by the private sector [26]. On-going efforts could be supported with mystery shoppers to check that providers are only stocking first-line anti-malarials, and be complemented by increased supervision and regulation to enforce removal of NTG anti-malarials from the market. In fact, a targeted private-sector intervention in the Lao PDR involving inspections of the pharmacies, provision of information on essential medicines, and distribution of malaria case management documents found marked improvements in the availability of essential medicines and concluded that these activities were an important factor behind the service quality improvements [37].

### Malaria diagnosis

#### Availability of any test and market share

Across outlet types and countries, private-sector availability of confirmatory testing among anti-malarial stocking outlets was generally moderate. However, gaps in testing availability were observed among outlets authorized to test, such as private-for-profit health facilities and pharmacies across all countries. In Myanmar, availability was particularly low. Closing gaps in availability of testing and first-line treatment in private sectors across the GMS is needed to ensure that people seeking treatment in the private sector are managed according to national guidelines.

Outlet survey methods do not allow for determining whether anti-malarials were distributed for confirmed or unconfirmed cases. However, the audit methodology can be used to summarize what proportion of anti-malarials distributed were dispensed by outlets with testing available to the patient. In Cambodia, anti-malarial distribution in both the public and private sectors was typically occurring in outlets that have confirmatory testing available, which is promising and suggests that testing is at least available to patients prior to being administered treatment. However, in the Lao PDR and Myanmar, the private sector appears to be a source for presumptive treatment. Most anti-malarials were distributed by private-sector outlets without confirmatory testing available. This is of particular concern in Myanmar, given that patients should be given different first-line treatment regimens according to whether or not they test positive for *P. falciparum* or *P. vivax.* As most anti-malarial distribution occurred through outlets that do not have testing available suggests that presumptive treatment is rife. Furthermore, while availability of first-line treatments was high in Myanmar, patients were unlikely to be treated correctly in the absence of confirmatory testing. As the NTGs are different for *P. falciparum* and *P. vivax* malaria, adhering to national malaria treatment guidelines was inherently impossible for most private-sector providers in the absence of confirmatory testing. Moving forward, closing these gaps will be key. Increased coverage of malaria testing  in the private sector will not only be important to ensure appropriate treatment and rational drug use, but also to track all confirmed cases towards a complete national surveillance system.

#### RDT availability with global quality-assured standards

The Global Fund to fight AIDS, Tuberculosis and Malaria (GFATM) periodically publishes a list of quality-assured RDT that are recommended for use after technical evaluation by the WHO Malaria RDT Testing programme and/or WHO Prequalification of Diagnostics programme [38]. Although RDT on this list have been shown to be accurate and reliable, inclusion does not preclude manufacturing quality failures or degradation due to prolonged storage or extreme conditions. Furthermore, RDT that have not been submitted for testing or prequalification may still meet regulatory standards and are eligible for procurement by GF principle recipients. Nonetheless, the GF quality assurance status is viewed as a global standard for RDT quality.

Most RDT-stocking outlets were stocking quality-assured RDT. However, non quality-assured RDT were available in 1 in 4 private-sector outlets in Cambodia and 1 in 5 private-sector outlets in Myanmar. Assured product quality is important given the variation in RDT brand performance. In the context of malaria elimination settings, it is imperative that future procurement of RDT includes only quality-assured products. Non quality-assured RDT can also leave providers uncertain about the reliability of results, which in turn can lead to the over-use of malaria medicines. However, with over 60 manufacturers of malaria RDT, this profusion can make it difficult for national malaria control programmes to determine which test or tests to purchase [39]. Continued guidance will be required to assist private-sector procurement services and private importers to take into account the quality-assurance status of RDTs when making purchasing and regulatory decisions [40].

### Coverage of private sector engagement and support

Key strategies to ensure that private-sector providers are contributing to national goals have included training, supervision, providing access to free or subsidized commodities, and ensuring that the private sector contributes caseload data to national surveillance systems. As previously discussed, these strategies are being implemented in various forms across the region, including the PPM programmes in Cambodia and the Lao PDR. In these countries, private-sector engagement targets providers in private for-profit health facilities and pharmacies. In Myanmar, various strategies are targeted to engage all private sector outlet types, including general retailers and itinerant drug vendors.

Results from these outlet surveys show that strategies implemented in each country were not reaching the majority of private providers. Fewer than half of providers reported receiving any sort of supervisory or regulatory visit within the past year and/or reporting malaria caseload data to government or non-governmental organizations. Less than half of the private providers in Cambodia, the Lao PDR reported access to subsidized ACT medicines and/or RDT, despite efforts across all three countries to increase access to subsidized anti-malarials through national private-sector distribution schemes. Supervision and caseload reporting were highest in the Lao PDR and were the result of the current PPM programme. The results from this study point to the need to scale-up up current PPM programmes in these countries, to include more licensed providers in the scheme as a means to ensure universal coverage of testing and treatment and increased private-sector regulation. Of promise is that national strategic plans for malaria elimination in both countries include scale-up and expansion of the PPM programme [20, 41]. These results may also serve as a baseline for the much-needed work of engaging private providers to ensure appropriate malaria case management and surveillance.

Several lessons can be taken from other pilot initiatives implemented to leverage the private sector in order to improve access to anti-malarials and RDT to improve malaria case management. Perhaps most noteworthy was the affordable medicines facility malaria (AMFm), which was designed to increase access to affordable quality-assured ACT medicines in both the public and private sectors in eight countries. During the AMFm pilot, manufacturers were provided copayments to subsidize wholesale quality-assured ACT medicine prices, and supportive interventions, including provider training and behaviour change communications were implemented. An evaluation of the AMFm pilot showed substantial increases in availability and market share for first-line treatment for uncomplicated malaria in most countries [42]. This was attributed to the subsidy but also several supportive interventions designed to create awareness and demand among patients and providers. Future strategies in the GMS designed to maximize coverage of the first-line treatment may want to consider scaling up supportive interventions to drive provider awareness for testing and first-line anti-malarial medicines as well as improving access to subsidized first-line treatments.

Improved private-sector malaria case reporting will also be important not only so that patients can be tracked and managed appropriately but also to ensure complete and timely case reporting [1]. This is especially important in elimination settings such as the GMS, where all cases must be tracked and investigated. Results from this study indicate that the majority of private-sector providers engaging in malaria testing and treatment do not provide malaria caseload data to a government authority or non-governmental organization across countries. Several challenges have been identified with incorporating the private sector into malaria surveillance systems [15]. Several initiatives are underway in Cambodia, the Lao PDR, and Myanmar to improve private-sector case management practices and ensure caseload reporting as part of the GMS Elimination of Malaria through Surveillance (GEMS) project in Cambodia, the Lao PDR, Myanmar, and Vietnam. The GEMS project includes provider training and supervision and strategies to strengthen private sector surveillance [43]. Caseload data from the private sector will be integrated with public-sector data to provide national programmes with a more complete picture of malaria burden and information to respond to all detected cases.

### Strengths and limitations

The ACTwatch project has conducted approximately 50 outlet surveys in 12 countries in Africa and Asia. The outlet survey methodology is rigorous and uses standardized methods and data collection tools across countries and over time. The use of a full census of all outlets with the potential to stock anti-malarials and/or malaria RDT allows for a unique analysis of the total malaria testing and treatment market. The surveys conducted in the GMS provide useful information that can be used to support national policy towards malaria control and elimination.

Several limitations should be considered when interpreting ACTwatch outlet survey results [16, 19], as with other medicine surveys [44]. This includes the cross-sectional nature of the surveys, potentially biased or misreported information, and challenges related to standardizing anti-malarial volumes and prices for products with different active ingredients and formulations. Providers may hide anti-malarials, especially providers who are not authorized to provide testing and treatment (e.g. general retailers), or providers who stock banned anti-malarials that are illegal to distribute (e.g. oral artemisinin monotherapy). This may be more common in malaria elimination settings where more resources are earmarked for enforcement of regulation [44]. Mystery client surveys may be an important source of data to triangulate results from the outlet surveys, particularly with respect to stocking practices of banned products when total market data is not needed. Finally, the outlet surveys conducted in the GMS were designed to generate results for key indicators including malaria testing and treatment availability, market share, and private-sector engagement and support and were not intended to evaluate specific programmes such as the PPM initiatives in the Lao PDR and Cambodia. Specific study designs with appropriate sampling techniques would be needed to determine the effectiveness and impact of these interventions.

## Conclusion

Findings from this multi-country study suggest that the private sector for malaria case management in Cambodia, Myanmar and the Lao PDR is generally in alignment with national regulations, treatment guidelines, and RDT quality-assurance standards. However, important gaps persist and pose a threat to national malaria control and elimination goals. In 2015, there was still a substantial amount of treatment not included in the NTGs stocked and distributed in Cambodia and Myanmar, as well as inappropriately high distribution of second-line *P. vivax* malaria treatment in the Lao PDR. As malaria elimination efforts intensify, it will be important to enforce removal of these products from private-sector outlets and to ensure only authorized outlets are providing malaria testing and treatment services. Decisions about whether or not informal providers warrant inclusion into the formal health system need to be made. Private-sector engagement was inadequate in Cambodia, the Lao PDR, and Myanmar. Increased engagement has the potential to improve malaria case management, readiness, and case reporting, and is thus critical for continued progress towards malaria elimination goals in the GMS.

## Additional files

**Additional file 1.** Availability of any anti-malarial among all outlets screened.

**Additional file 2.** Catalogue of anti-malarials found in the private sector that were not indicated in the national treatment guidelines.

## Abbreviations

ACT: Artemisinin-based combination therapy
AETD: adult equivalent treatment dose
AMFm: affordable medicines facility malaria
AMTR: Artemisinin Monotherapy Replacement Project
GEMS: GMS Elimination of Malaria through Surveillance
GF: Global Fund to fight AIDS, Tuberculosis and Malaria
GMP: good manufacturing practices
GMS: Greater Mekong Subregion
HMIS: Health Management Information System
MoH: Ministry of Health
NTG: National Treatment Guidelines
PCN: product catalogue number
PPM: public–private mix
PSI: Population Services International
PPS: probability proportional to size
PSK: Population Services Khmer
RDT: rapid diagnostic test
WHO: World Health Organization
