# Supplementary material for: Private sector opportunities and threats to achieving malaria elimination in the Greater Mekong Subregion: results from malaria outlet surveys in Cambodia, the Lao PDR, Myanmar, and Thailand
Source: Malar J. 2017 May 2;16:180. doi: 10.1186/s12936-017-1800-5 (PMC5414126; doi:10.1186/s12936-017-1800-5)
Supplement: Supplementary file 1 — Additional file 1. Availability of any anti-malarial among all outlets screened. [file 12936_2017_1800_MOESM1_ESM.docx]

*In Myanmar, drug stores are excluded given on 15 cases were audited
