# Supplementary material for: Private sector opportunities and threats to achieving malaria elimination in the Greater Mekong Subregion: results from malaria outlet surveys in Cambodia, the Lao PDR, Myanmar, and Thailand
Source: Malar J. 2017 May 2;16:180. doi: 10.1186/s12936-017-1800-5 (PMC5414126; doi:10.1186/s12936-017-1800-5)
Supplement: Supplementary file 2 — Additional file 2. Catalogue of anti-malarials found in the private sector that were not indicated in the national treatment guidelines. [file 12936_2017_1800_MOESM2_ESM.docx]

| **Cambodia** |
| --- |

| Generic | Formulation | Brand | Manufacturer | Country of Manufacturer | Nationally Registered | Number audited | | Outlet type | | | |  |
| --- | --- | --- | --- | --- | --- | --- | --- | --- | --- | --- | --- | --- |
|  |  |  |  |  |  |  |  | HF | PH | DS | GR | IV |
| Artemisinin piperaquine | Tablet | Artequick | Artepharm | China | No | 31 | **×** | **×** | **×** | **×** | **×** |  |
| Artesunate | Tablet | Artesunate | Binhdinh | Vietnam | No | 1 |  |  |  | **×** |  |  |
| AS MQ | Tablet | A + M4 | Cipla Pharma | India | No | 1 |  |  |  | **×** |  |  |
| AS MQ | Tablet | A + M5 | Cipla Pharma | India | No | 5 | **×** |  |  |  | **×** |  |
| AS MQ | Tablet | Malarine - adults | Cipla Pharma | India | No | 13 | **×** | **×** | **×** | **×** | **×** |  |
| AS MQ | Tablet | Malarine - children | Cipla Pharma | India | No | 3 |  | **×** |  |  | **×** |  |
| AS MQ | Tablet | Malarine - teenagers | Cipla Pharma | India | No | 5 |  | **×** | **×** |  | **×** |  |
| Chloroquine | Tablet | Chloroquine | Acdhon | Thailand | No | 33 | **×** | **×** | **×** | **×** | **×** |  |
| Chloroquine | Tablet | Nitaquine | Utopian | Thailand | No | 9 | **×** |  | **×** | **×** | **×** |  |
| Chloroquine | Tablet | Choroquine | Unknown | Unknown | No | 25 |  |  | **×** | **×** | **×** |  |

| **Lao PDR** | | | | | | | | | | | |  |
| --- | --- | --- | --- | --- | --- | --- | --- | --- | --- | --- | --- | --- |
| Generic | Formulation | Brand | Manufacturer | Country of Manufacturer | Registered | Number audited | | Outlet type | | | |  |
|  |  |  |  |  |  |  |  | HF | PH | DS | GR | IV |
| Artesunate | Tablet | Artesunat | Armephaco | Vietnam | No | 1 | **×** |  |  |  |  |  |
| Chloroquine | Injection | Malacin | ANB Laboratories | Thailand | Yes | 45 | **×** | **×** | **×** |  |  |  |
| DHA PPQ | Tablet | CV Artecan | OPC Pharmaceutical | Vietnam | No | 1 | **×** |  |  |  |  |  |
| Hydroxychloroquine | Tablet | HCQ 200 | Getz Pharma | Pakistan | No | 2 | **×** |  |  |  |  |  |

| **Myanmar** | | | | | | | | | | | |
| --- | --- | --- | --- | --- | --- | --- | --- | --- | --- | --- | --- |
| Generic | Formulation | Brand | Manufacturer | Country of Manufacturer | Registered | Number audited | Outlet type | | | | |
|  |  |  |  |  |  |  | HF | PH | DS | GR | IV |
| Arteether | Injection | Arteether | Shivek Labs | India | No | 3 |  | **×** |  | **×** |  |
| Artemether | Tablet | AA-Artemether | AA Medical Products | Vietnam | No | 22 | **×** | **×** |  | **×** |  |
| Artemether | Tablet | Artem | Kunming Pharmaceutical | China | No | 23 | **×** | **×** |  | **×** | **×** |
| Artemether | Tablet | Artemether | Chongging Holley Pharmaceutical | China | No | 6 | **×** | **×** |  |  | **×** |
| Artemether | Tablet | Artemether | Kunming Pharmaceutical | India | No | 13 |  | **×** |  | **×** |  |
| Artemether | Tablet | Artemether | Unknown | Unknown | No | 13 | **×** | **×** |  | **×** |  |
| Artesunate | Tablet | AA-Artesunat | AA Medical Products | Vietnam | No | 13 | **×** | **×** |  | **×** | **×** |
| Artesunate | Tablet | Artesunate | Central Pharmaceutical Factory No. 1 | Vietnam | No | 2 | **×** | **×** |  |  |  |
| Artesunate | Tablet | Artesunate | Jiangxi Xierkangtai Pharmaceutical | China | No | 1 | **×** |  |  |  |  |
| Artesunate | Tablet | Artesunate | Mediplantex | Vietnam | No | 784 | **×** | **×** |  | **×** | **×** |
| Artesunate | Tablet | Artesunate | Medopharm | Vietnam | No | 2 |  |  |  | **×** |  |
| Artesunate | Tablet | Artesunate | Mekophar | Vietnam | No | 1 |  | **×** |  |  |  |
| Artesunate | Tablet | Artesunate | Myanmar/Tatmadaw Pharmaceutical Factory | Myanmar | No | 12 | **×** | **×** |  | **×** | **×** |
| Artesunate | Tablet | Artesunate | Zhangfeng Pharmaceutical Factory | China | No | 17 |  | **×** |  | **×** |  |
| Artesunate | Tablet | Arthesis | Zhejiang Holley Nanhu Pharmaceutical | China | No | 10 | **×** | **×** |  | **×** | **×** |
| Artesunate | Tablet | Traphasunat | Traphaco | Vietnam | No | 9 | **×** | **×** |  | **×** |  |
| Artesunate | Tablet | Artesunate | Unknown | Unknown | No | 40 | **×** | **×** |  | **×** | **×** |
| Artemisinin napthoquin | Tablet | Arco | Kunming Pharmaceutical | China | No | 1 |  |  |  |  | **×** |
| Artesunate amodiaquine | Tablet | Artemodi | Jiaxing Nanhu Pharmaceutical | China | No | 1 |  |  |  |  | **×** |

| **Myanmar** | | | | | | | | | | | |
| --- | --- | --- | --- | --- | --- | --- | --- | --- | --- | --- | --- |
| Generic | Formulation | Brand | Manufacturer | Country of Manufacturer | Registered | Number audited | Outlet type | | | | |
|  |  |  |  |  |  |  | HF | PH | DS | GR | IV |
| Chloroquine | Injection | Malacin | ANB Laboratories | Thailand | No | 6 |  | **×** |  | **×** | **×** |
| Mefloquine | Tablet | Mefloquine | Myanmar/Tatmadaw Pharmaceutical Factory | Myanmar | No | 17 | **×** | **×** |  | **×** | **×** |
| Mefloquine | Tablet | Mephaquin | Mepha | Switzerland | Yes | 1 |  | **×** |  |  |  |
| Mefloquine | Tablet | Mefloquine | Unknown | Unknown | No | 7 | **×** | **×** |  | **×** | **×** |
| Quinine | Tablet | Quinine | Myanmar/Tatmadaw Pharmaceutical Factory | Myanmar | No | 32 | **×** | **×** |  | **×** | **×** |
| Quinine | Tablet | Quinine | Unknown | Unknown | No | 8 |  | **×** |  | **×** | **×** |
| SP | Tablet | Pyrixine | Myanmar/Tatmadaw Pharmaceutical Factory | Myanmar | No | 66 | **×** | **×** |  | **×** | **×** |

| **Thailand** | | | | | | | | | | | |
| --- | --- | --- | --- | --- | --- | --- | --- | --- | --- | --- | --- |
| Generic | Formulation | Brand | Manufacturer | Country of Manufacturer | Registered | Number audited | Outlet type | | | | |
|  |  |  |  |  |  |  | HF | PH | DS | GR | IV |
| Atovaquone proguanil | Tablet | Malanil | GlaxoSmithKline | Canada | Yes | 1 | **×** |  |  |  |  |
| Hydroxychloroquine | Tablet | Hydroquin | Sun Pharmaceutical Industries | India | No | 2 | **×** |  |  |  |  |
| Mefloquine* | Tablet | Mequin | Atlantic Laboratories | Thailand | Yes | 3 | **×** |  |  |  |  |

* In Thailand, AS MQ is the first-line treatment for *Pf* malaria, however, this mefloquine product was audited in an outlet without artesunate tablets so was classified as mefloquine monotherapy
